# Loss of *Ccbe1* affects cardiac-specification and cardiomyocyte differentiation in mouse embryonic stem cells

**DOI:** 10.1371/journal.pone.0205108

**Published:** 2018-10-03

**Authors:** Oriol Bover, Tiago Justo, Paulo N. G. Pereira, João Facucho-Oliveira, José M. Inácio, José S. Ramalho, Ibrahim J. Domian, José António Belo

**Affiliations:** 1 Stem Cells and Development Laboratory, CEDOC, Chronic Diseases Research Centre, NOVA Medical School|Faculdade de Ciências Médicas, Universidade NOVA de Lisboa, Lisboa, Portugal; 2 Center for Biomedical Research, Campus de Gambelas, University of Algarve, Faro, Portugal; 3 CEDOC, Chronic Diseases Research Centre, NOVA Medical School|Faculdade de Ciências Médicas, Universidade NOVA de Lisboa, Lisboa, Portugal; 4 Cardiovascular Research Center, Massachusetts General Hospital, Boston, Massachusetts, United States of America; 5 Harvard Stem Cell Institute, Cambridge, Massachusetts, United States of America; Laboratoire de Biologie du Développement de Villefranche-sur-Mer, FRANCE

## Abstract

Understanding the molecular pathways regulating cardiogenesis is crucial for the early diagnosis of heart diseases and improvement of cardiovascular disease. During normal mammalian cardiac development, collagen and calcium-binding EGF domain-1 (*Ccbe1*) is expressed in the first and second heart field progenitors as well as in the proepicardium, but its role in early cardiac commitment remains unknown. Here we demonstrate that during mouse embryonic stem cell (ESC) differentiation *Ccbe1* is upregulated upon emergence of *Isl1*- and *Nkx2*.*5*- positive cardiac progenitors. *Ccbe1* is markedly enriched in *Isl1*-positive cardiac progenitors isolated from ESCs differentiating *in vitro* or embryonic hearts developing *in vivo*. Disruption of *Ccbe1* activity by shRNA knockdown or blockade with a neutralizing antibody results in impaired differentiation of embryonic stem cells along the cardiac mesoderm lineage resulting in a decreased expression of mature cardiomyocyte markers. In addition, knockdown of *Ccbe1* leads to smaller embryoid bodies. Collectively, our results show that CCBE1 is essential for the commitment of cardiac mesoderm and consequently, for the formation of cardiac myocytes in differentiating mouse ESCs.

## Introduction

Identification of genes and the study of their role in cardiogenesis are important to elucidate the molecular events regulating cardiomyocyte lineage commitment. This is critical for the control of cardiac commitment from different stem cell sources and the use of mature cardiac cells in the context of regenerative medicine. In a differential screen designed to identify novel genes required for the correct development of the heart precursor lineages [1], we identified *CCBE1*, a gene coding for a secreted protein that contains collagen domains and a calcium binding EGF-like domain. Expression analysis showed that *Ccbe1* is expressed in precursors of the first heart field (FHF), secondary heart field (SHF), and proepicardium in mice between embryonic day (E) 7.0 to E9.5 [2]. Similarly, *CCBE1* was similarly found to be expressed in FHF and SHF populations during early chick cardiac development [3]. These findings implicate CCBE1 in the control of early cardiac commitment, but its function in this context remains elusive. Previous work has also shown that *Ccbe1* is expressed in the pericardium between E11.0 and E12.5 [4], however, at these stages *Ccbe1* is deeply involved in the development of the lymphatic system. Indeed, *Ccbe1* loss-of-function in mice leads to prenatal death due to defective lymphatic vasculature [4]. *Ccbe1* is required for the budding and migration of lymphatic endothelial cells (LECs) from the anterior cardinal veins to give rise to the lymphatic vasculature [4, 5]. Absence of proper lymphatic vessels results in generalized tissue edema by E14.5 and the death of mutant embryos shortly after. Another report also demonstrates that absence of the collagen domains from CCBE1 in mice fully phenocopies the *null* mutant [6]. The mode of action of CCBE1 involves the recruitment of the metalloprotease ADAMTS3 extracellularly to promote the conversion of immature (Pro-)VEGF-C into its mature and fully active pro-lymphangiogenic form [7, 8].

In humans, mutations in CCBE1 have been associated with Hennekam syndrome (HS), a disorder characterized by abnormal lymphatic system development. Interestingly, some patients also present with congenital heart defects including hypertrophic cardiomyopathy and ventricular septal defects [9–11], consistent with a role of CCBE1 during heart formation. Although two recent studies suggest that cardiac development is normal in *Ccbe1* mutant mice [12, 13], we showed that *CCBE1* is required for the migration of the cardiac precursor cells to form the heart tube during chicken heart development [3]. Modulation of *CCBE1* levels in the chick embryos leads to cardia bifida when the cardiac fields are exposed to high levels of *CCBE1*. Conversely, exposure to low levels of *CCBE1* result in incorrect fusion of the bilateral cardiac fields to form the heart tube. Therefore, given those opposing observations about the role of CCBE1 in the development of the heart from different species, we sought to study the role of CCBE1 during cardiogenesis using an established model of cardiac differentiation *in vitro* using mouse ESCs.

Here, we analyze the effect of *Ccbe1* loss-of-function during differentiation of mouse ESCs and identify a role in early cardiac mesoderm commitment as well as in cell proliferation. In addition, we examine *Ccbe1* expression in differentiating mouse ESCs and confirm its expression in isolated cardiac progenitor populations derived from ESCs.

## Materials and methods

### Culture of mouse ESCs

Nkx2.5-GFP/SHF-dsRed (RG) mouse ESCs [14] were cultured in knockout Dulbecco's Modified Eagle Medium (DMEM, Sigma) with 15% Fetal Bovine Serum (FBS, Hyclone, Utah, US), 1% penicillin/streptomycin solution (Life Technologies), 2 mM L-glutamine (Life Technologies), 1% non-essential aminoacids (Life Technologies), 0.1 mM-mercaptoethanol (Sigma) and 1000 U/mL leukemia inhibitory factor (LIF; Chemicon, Temecula, Ca, USA). Mouse ESCs were cultured in 0.1% gelatin coated dishes at 37°C/5%CO_2_.

### Differentiation and culture of mouse ESCs by hanging droplet method

RG mouse ESCs were differentiated using the hanging droplet method [15]. In short, undifferentiated mouse ESCs were resuspended in differentiation medium, consisting of mouse ESCs medium without LIF. Approximately 500 ESCs were used per droplet and cells were cultured in hanging droplets for 2 days to allow the formation of embryoid bodies. Embryoid bodies were then cultured in static suspension culture until day 5 of differentiation, followed by adherent culture in gelatin (0.1%) coated wells at a density of 12 embryoid bodies per well of a 6 well plate up to day 10 of differentiation. For the experiments using CCBE1 antibody supplementation as a blocking antibody, ESCs were differentiated until day 5 in the presence of 100 ng/mL of anti-CCBE1 antibody (Abcam, ab101967) in the differentiation medium.

### Generation and analysis of *Ccbe1* KD mouse ESCs

Transgenic mouse ESC lines expressing control scrambled and *Ccbe1-*targeting shRNAs were generated using commercially available shRNA control and two Ccbe1-shRNA lentiviral plasmids (Sigma, SHC002, TRCN0000091779, and TRCN0000091781), respectively. Lentiviral particles were produced in HEK293T cells according to manufacturer’s instructions. RG mouse ESCs were infected with the viral particles, selected using puromycin (Sigma) and individual clones picked to isolate stable scrambled (SH) control and *Ccbe1* knockdown (KD) ESC lines. Knockdown was assessed by comparing *Ccbe1* expression by quantitative PCR (qPCR) from the parental (WT) RG mouse ESC line with the individual clones of the SH control ESC line and *Ccbe1* KD ESC lines. Selected clones of the transgenic *Ccbe1* KD ESC lines and the control mouse ESC line were allowed to differentiate *in vitro* as described above. Samples were collected from embryoid bodies at several days of differentiation as specified for each experiment for qPCR and fluorescent cell sorting analysis (FACS). Three biological replicates were considered for the analysis of the results.

### RNA isolation, cDNA synthesis and qPCR

Total RNA was extracted using TRI Reagent (Sigma) and the Direct-zol RNA MiniPrep kit (Zymo Research) following the manufacturer’s instructions. RNA concentration was determined using NanoDrop ND- 2000c Spectrophotometer (NanoDrop Technologies) and integrity confirmed using the Experion RNA electrophoresis system (Bio-Rad). Reverse transcription was performed using RevertAid Reverse Transcriptase, oligo-dT primer, RiboLock RNAse Inhibitor and dNTPs (Thermo Fisher Scientific). qPCR was performed on 7300 Real-Time PCR System (Applied Biosystems) using Power SYBR Green PCR Master Mix (Applied Biosystems). Primers for the genes of interest (*Ccbe1*, *Mesp-1*, *Isl1*, *Nkx2*.*5*, *cTnt* and *αMhc*) are presented in S1 Table. *Gapdh* and *Pgk1* were used as reference genes. Data are expressed as mean ± SEM.

### Fluorescent-activated cell sorting (FACS)

RG mouse ESCs were sorted on a Becton Dickinson FACSAria II (BD Biosciences) using the FACSDiva 6.1.3 software (BD Biosciences). Flow cytometer was carried out upon excitation with the blue laser (488 nm) being the emission signals measured in the FL1 channel (530/30 nm) and in the FL2 channel (585/42 nm). Prior to FACS, samples were resuspended in PBS. Cells were ran through the FACS sorter to isolate the FHF progenitors (G^+^R^-^), the SHF progenitors (G^+^R^+^ and G^-^R^+^) and the control cells (G^-^R^-^).

### Isolation of embryonic cardiac progenitors

Embryos of Nkx2.5-GFP/SHF-dsRed double transgenic mice [14] were dissected at E9.5. Embryos were dissociated into single-cell by gentle trypsinization followed by passage through a 40 μM cell strainer. R^+^G^+^, R^-^G^+^ and R^+^G^-^ progenitors and R^-^G^-^ controls were isolated by FACS as described above, collected into TRIzol reagent and stored at -80°C prior to RNA isolation. Experiment was performed compliant with the US law and approved by the ethical commission from the *Massachusetts General Hospital*.

### Cell proliferation assay with Click-iT EdU

Newly synthesized DNA was labeled by incubating embryoid bodies with EdU for 1.5 hours. Subsequent EdU detection was done following the Click-iT EdU Alexa Fluor 488 Imaging Kit (Thermo Fisher Scientific) indications. Relative fluorescence intensity of cells was analyzed by Becton Dickinson FACSCanto II (BD Biosciences). Graphics were generate using FlowJo v10 software (BD). A minimum of 20,000 events were acquired for each ESC cell line.

### Cell apoptosis assay with cleaved caspase 3 immunolabelling

Cultured ESCs were trypsinized, resuspended on 3% FBS solution in PBS, and then centrifuged for 15 minutes at 4°C. Then, cells were fixed in 4% PFA for 20 minutes at RT. Cells were washed twice in blocking solution (1% BSA, 0,05% Triton in PBS) and left to incubate with polyclonal rabbit anti-activate caspase-3 primary antibody (R&D Systems, AF835; 1:500 dilution) for 30 minutes on ice. Cells were then washed twice in blocking solution and left to incubate with the secondary antibody in the dark for 25 minutes on ice. Following two washes with blocking solution, cells were resuspended in PBS and analyzed by FACS as described before.

### CCBE1 blockade with antibody supplementation

WT RG ESCs were differentiated up to day 5 in the presence of CCBE1 antibody (Abcam, ab101967) at a concentration of 100 ng/mL diluted in the culture medium. The equivalent amount of buffer was used as a control.

### Statistics

Statistical analysis was performed using using GraphPad Prism version 5.00 for Windows, GraphPad Software, San Diego California USA. In the qPCR analysis, for the comparisons between differentiating ESC lines we used paired student’s t-test. Differences were considered significant when * p<0.05, ** p<0.01 and *** p<0.001.

## Results

### High *Ccbe1* expression coincides with the appearance of cardiac progenitors

To evaluate *Ccbe1* expression during cardiac differentiation, we exploited the double transgenic RG ESC line, wherein the red fluorescent protein dsRed is under the control of the second heart field (SHF) enhancer of *Mef2C*, and the enhanced green fluorescent protein (GFP) is under the control of the cardiac-specific enhancer of *Nkx2*.*5* [14]. This ESC line allows for the isolation of pure populations of FHF (GFP^+^/dsRed^-^ or G^+^R^-^) and SHF (GFP^+^/dsRed^+^ or G^+^R^+^, and GFP^-^/dsRed^+^ or G^-^R^+^) progenitors by FACS.

**Fig 1:**
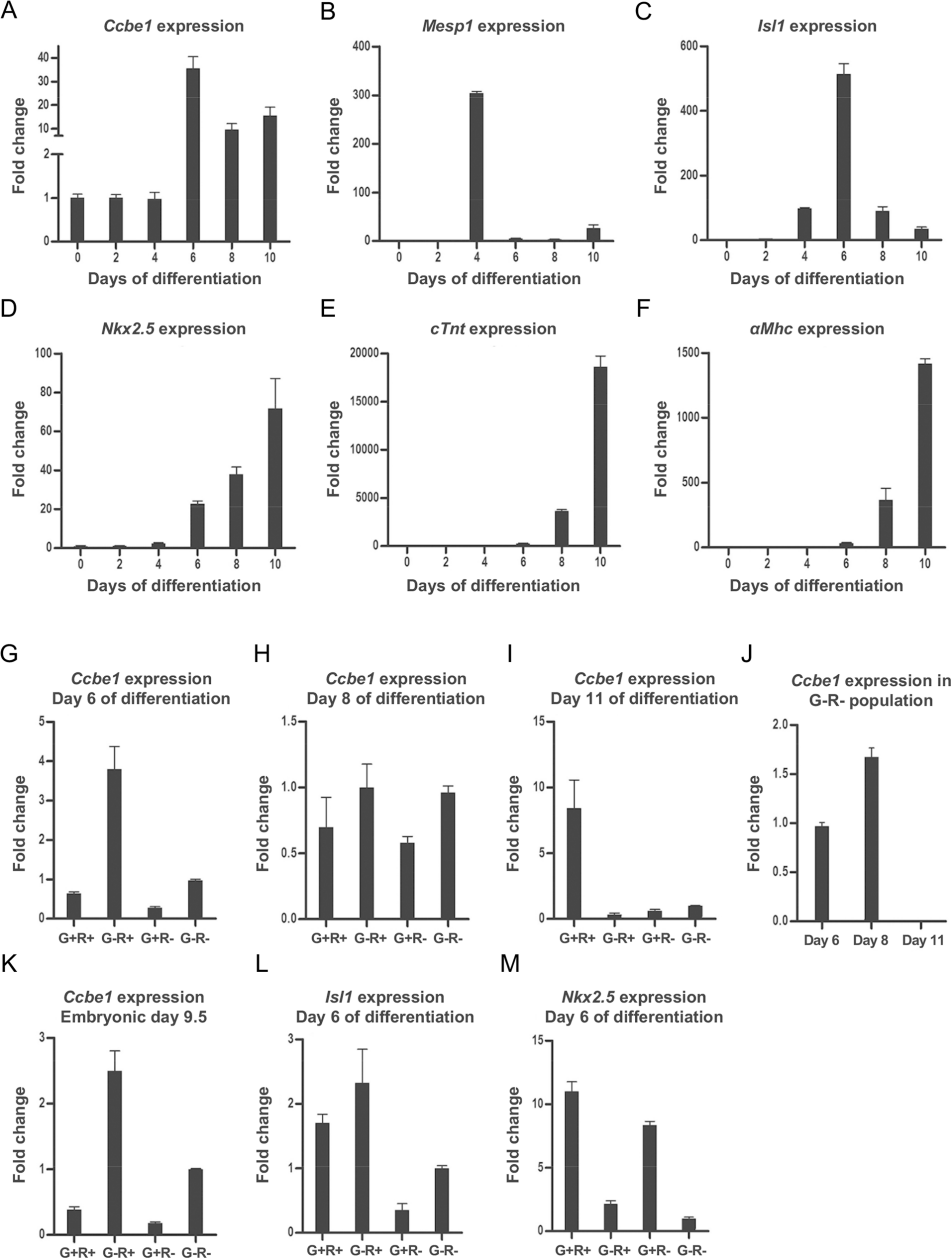
Expression of markers of interest during differentiation of mouse ESCs Ccbe1 in cardiac progenitors isolated from differentiating mouse ESCs and embryos at E9.5. (A-F) Samples were collected from undifferentiated cells (Und) and at days 2, 4, 6, 8 and 10 of differentiating RG mouse ESCs. Expression is presented as fold change relative to undifferentiated cells. Data presented as the mean + SEM of two biological replicates in technical qPCR triplicates. (G-M) *Ccbe1* expression was analyzed in isolated populations, defined as FHF G+R- population, SHF G+R+ and G-R+ populations and control G-R- population at (G) day 6, (H) day 8 and (I) day 11 from differentiating mouse ESCs, and from (K) E9.5 mouse embryos. Expression is represented as fold change relative to the control G-R-population. (J) Analysis of *Ccbe1* expression in the control G-R- population at day 6, 8 and 11 relative to the expression at day 6. The identity of the sorted populations at day 6 was confirmed by analyzing the expression of (L) Ist1and (M) Nkx2.5. Mean + SEM of two biological replicates.

Mouse RG ESCs were differentiated as embryoid bodies using the hanging droplets method and samples collected every 48 hours to examine *Ccbe1* expression by quantitative PCR (qPCR). Cardiac specific genes including *Mesp-1*, *Isl1*, *Nkx2*.*5*, *αMhc* and *cTnt*, were then assayed as markers of cardiac differentiation. Expression analysis showed high levels of *Mesp-1* at day 4 of differentiation (Fig 1B) consistent with the formation of cardiac mesoderm. The expression of the cardiac mesoderm and SHF marker *Isl1* increased considerably at day 4 and peaked at day 6, while the general cardiac progenitors and primitive cardiomyocyte marker *Nkx2*.*5* increased only at day 6 (Fig 1C and 1D). The appearance of *Isl1* expression at day 4, prior to *Nkx2*.*5*, is consistent with the *in vivo* situation where *Isl1* expression precedes *Nkx2*.*5* expression during the formation of cardiac mesoderm [16]. The cardiomyocyte markers *cTnt* and *αMhc* were first expressed at day 6 with peak expression at day 10 (Fig 1E and 1F). Analysis of *Ccbe1* expression revealed similar levels of *Ccbe1* in undifferentiated mouse ESCs and days 2 and 4 of differentiation (Fig 1A). Higher *Ccbe1* expression was detected at day 6 of differentiation, concurrent with peak of expression of cardiac progenitor marker *Isl1* and the increasing expression of *Nkx2*.*5*. At days 8 and 10, *Ccbe1* was highly expressed but not as high as day 6. This suggests that in embryonic stem cells differentiating *in vitro*, like mouse embryos developing *in vivo* [2], the appearance of high levels of *Ccbe1* expression coincide with the formation of FHF and SHF cardiac progenitors.

### *Ccbe1* is expressed in SHF and proepicardium cardiac progenitors

In order to confirm that the increase in *Ccbe1* expression is associated with the formation of FHF (G^+^R^-^) and SHF (G^+^R^+^ and G^-^R^+^) cardiac progenitor cells, we isolated these populations by FACS and analyzed *Ccbe1* expression along with *Isl1* and *Nkx2*.*5* expression to confirm the identity of the isolated populations. Expression analysis revealed *Ccbe1* transcripts in all cardiac progenitor populations (Fig 1G–1I). At day 6, *Ccbe1* expression was higher (3.8 fold) in SHF G^-^R^+^ cardiac progenitor cells or SHF-derived cells compared to the double negative control (Fig 1G). In contrast, *Ccbe1* expression was down regulated in SHF G^+^R^+^ and FHF G^+^R^-^ cardiac progenitors when comparing to the same control population. Interestingly, a very similar *Ccbe1* expression profile was observed in FHF and SHF progenitors isolated from mouse embryos at E9.5 (Fig 1K). *Ccbe1* was also enriched in the equivalent SHF G^-^R^+^ population isolated from Nkx2.5-GFP/SHF-dsRed transgenic mouse embryos (2.5 fold). *Isl1* and *Nkx2*.*5* expression is consistent with the identity of the sorted populations from mouse RG ESCs at day 6 of differentiation (Fig 1L and 1M).

At day 8 of ESC differentiation, the G^-^R^+^ cells continue to have higher *Ccbe1* expression than the G^+^R^+^ and G^+^R^-^ progenitors (Fig 1H). However, *Ccbe1* expression in the SHF G^-^R^+^ population was similar to the expression in control G^-^R^-^ population, likely due to the emergence of non-cardiac cell types (G^-^R^-^) that also express high levels of *Ccbe1* (Fig 1K). *Ccbe1* has been previously shown to be expressed in the dermamyotome of the somites and in cells located in the vicinity of the anterior cardinal veins between stages E8.75 and E10.5 mouse development [2]. At day 11, *Ccbe1* was mainly observed in the G^+^R^+^ population (Fig 1I). This is likely related with the formation of proepicardium progenitors, which are known to derive from *Nkx2*.*5*^+^/*Isl1*^+^ cells at the lateral zone of the cardiogenic mesoderm [17, 18] and were also shown to express *Ccbe1* in mouse embryos [2]. Taken together, these results show that *Ccbe1* expression in differentiating mouse ESCs is concurrent with the appearance of specific populations of cardiac progenitors.

### *Ccbe1* knockdown leads to reduced cardiac mesoderm formation from differentiating ESCs

**Fig 2:**
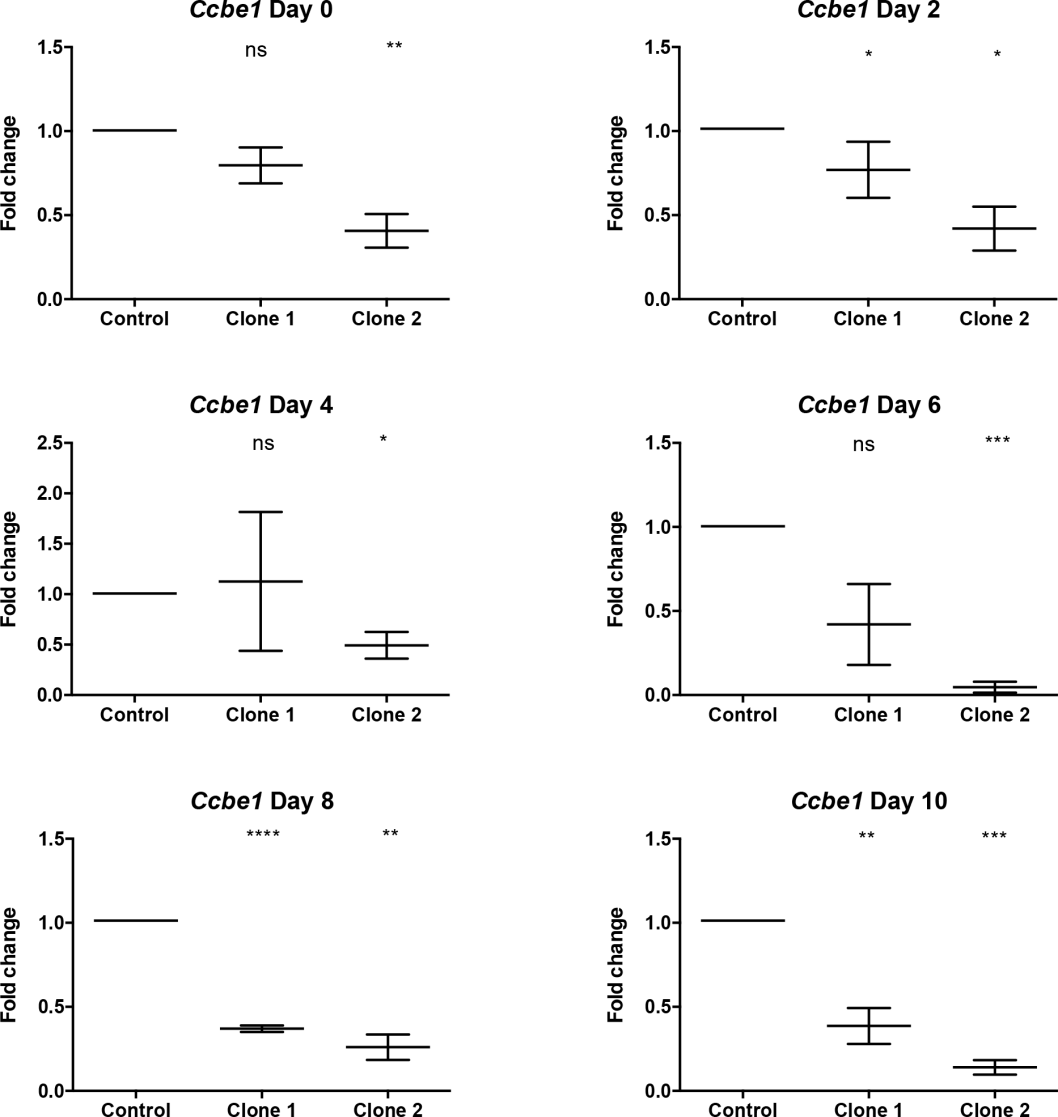
Expression of Ccbe1 during differentiation of Ccbe1 knockdown mouse ESC lines. Transgenic mouse ESC lines expressing control scrambled and Ccbe1-targeting shRNAs (Clone 1 and 2) were generated using commercially available shRNA control and two Ccbe1-targeted shRNA lentiviral plasmids. Selected clones of the transgenic Ccbe1KD ESC lines and the control mouse ESC line were allowed to differentiate. Samples were collected from undifferentiated cells (D0) and at days 2 (D2), 4 (D4), 6 (D6), 8 (D8) and 10 (D10) of differentiating clones. Expression is presented as fold change relative to the control of each day. Data presented as the mean + SEM of three biological replicates in technical qPCR triplicates.

The presence of *Ccbe1* expression early on in both mouse ESC derived and mouse embryonic cardiac progenitors led us to hypothesize that *Ccbe1* could have a role during establishment or maintenance of cardiac progenitors. To test this hypothesis, we generated two transgenic mouse ESC lines expressing shRNAs targeting *Ccbe1* and evaluated the effect of *Ccbe1* loss-of-function in spontaneous ESC-derived cardiac differentiation. *Ccbe1* KD ESC clonal lines were generated by lentiviral transduction using RG as WT line. We carried out spontaneous differentiation of one *Ccbe1* KD ESC clones for both shRNAs (Clone 1 and Clone 2) comparing it with the SH control ESC line to exclude off target effects of the shRNAs. Analysis of *Ccbe1* expression in undifferentiated cells confirmed significant *Ccbe1* downregulation in Clone 2 and partial downregulation in Clone 1 when compared to the *Ccbe1* levels found as in the control line (Fig 2A). During differentiation, Clone 2 exhibited significant reduction of *Ccbe1* expression at all days analyzed (2, 4, 6, 8, and 10). Clone 1 showed statistically significant reduction at days 2, 8 and 10 (Fig 2). Additionally, when *Ccbe1* expression normally peaks at day 6 of differentiation (Fig 1A), Clone 2 showed a very strong reduction in the expression of *Ccbe1* (Fig 2D) together with Clone 1, which displayed a visible reduction as well. Together, these data confirm that knockdown of *Ccbe1* was maintained during the *in vitro* differentiation, being this effect stronger in Clone 2.

**Fig 3:**
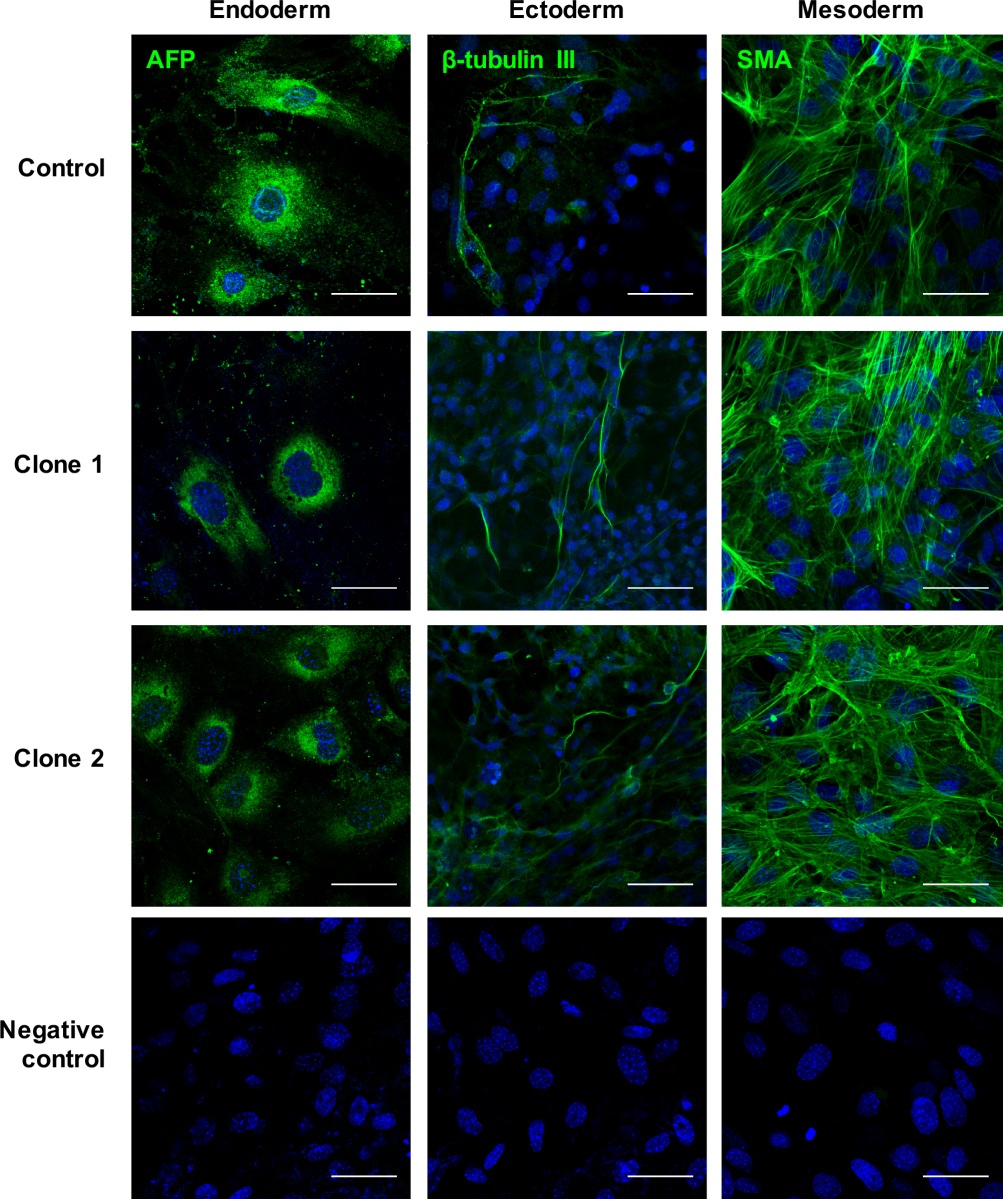
Analysis of expression of germ layer markers by immunofluorescence at day 10 of differentiation. shRNA control, Clone 1 and Clone 2 were let to differentiate until day 10. Differentiation into the three germ layers was analyzed by immunofluorescence of Alpha-fetoprotein (Endoderm), β-tubulin III (Ectoderm) and alpha smooth muscle actin (Mesoderm). Negative controls were only incubated with secondary antibody. Scale bar: 50 μm.

Analysis by immunofluorescence using confocal microscopy at day 10 showed that *Ccbe1* knockdown in these generated stable clones did not block its differentiation potential towards the three germ layers (Fig 3).

**Fig 4:**
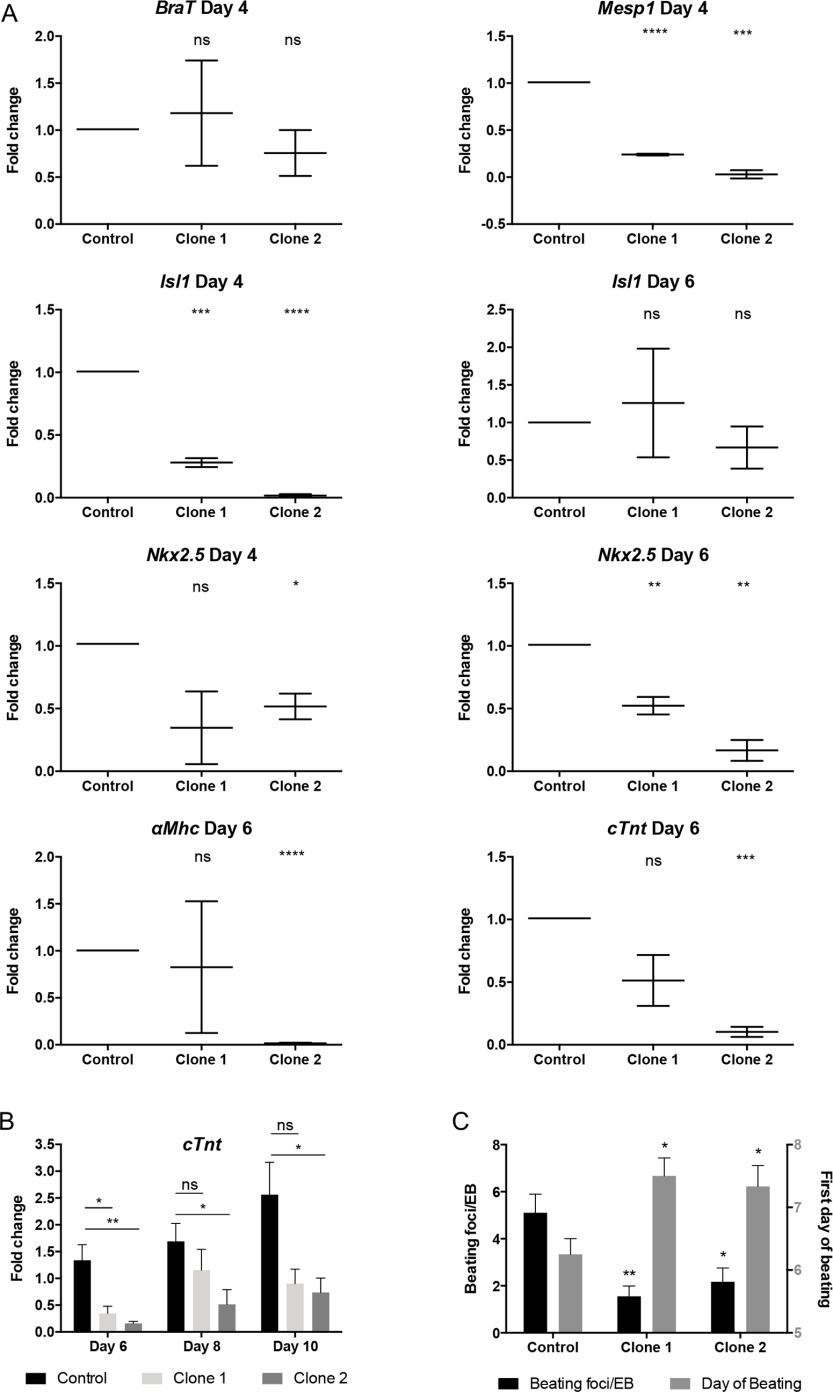
Ccbe1 knockdown leads to reduced cardiac mesoderm formation from differentiating mouse ESCs. (A) qPCR analysis of two individual Ccbe1 KD ESC clones (Clone 1 and Clone 2) compared to SH Control at Day 4 of the mesoderm marker brachyury (*BraT*) and cardiac precursor markers Mesp1, Isl1, and Nkx2.5; and at Day 6 of the cardiac markers *Isl1*, *Nkx2*.*5*, *cTnt* and *αMhc*. (B) qPCR analysis between Day 6 and 10 of the cardiomyocyte marker c*TnT*. All samples are relative to the control at Day 6. Mean ± SEM of three biological replicates in technical qPCR triplicates; unpaired t-test relative to Control; statistical significance *p < 0.05, **p < 0.01, ***p < 0.001, **** p < 0.0001. (C) Plated EBs were checked every day for beating and then analyzed at day 10 of differentiation. Beating foci were counted under the microscope. Mean ± SEM of at least three biological replicates; unpaired t-test relative to Control; statistical significance *p < 0.05, **p < 0.01, ***p < 0.001, **** p < 0.0001.

In agreement with this, expression of the early mesoderm marker *BraT* was not affected in differentiating SH control ESCs and both *Ccbe1* KD ESC lines (Fig 4A and S1 Fig). We then observed that expression of cardiac mesoderm markers *Mesp1* and *Isl1* was markedly reduced both in Clone 1 and Clone 2 at day 4 of differentiation (Fig 4A). In addition, expression of *Nkx2*.*5* was also reduced, in both differentiated *Ccbe1* KD clones. These data suggest that in the absence of *Ccbe1*, ESCs have reduced capacity to differentiate towards the *Mesp1* and *Isl1* cardiogenic mesoderm lineage. This in turn may result in a reduction of Nkx2.5-expressing cardiac progenitor cells, whose expression is specially reduced at day 6 of differentiation. In contrast, *Isl1* did not seem to be affected at this timepoint (Fig 4A). In the case of the early cardiomyocyte markers, *αMhc* and *cTnT*, Clone 2 showed statistically significant reduction whereas Clone 1 only showed a reduction in α*Mhc* but not statistically significant. This could indicate that differentiation towards mature cardiomyocytes is reduced in the absence of *Ccbe1*, namely in Clone 2 which showed consistent reduction throughout differentiation. A closer analysis between days 6 and 10 of differentiation shows a downregulation of the expression of c*TnT* which persists up until day 10 (Fig 4B). The number of beating foci was also significantly reduced on both clones at day 10 of differentiation, also, the EBs of both clones started to beat later (Fig 4C).

Together these data suggest that *Ccbe1* is essential for the formation of ESC-derived *Mesp1*- and *Isl1*-expressing cardiac mesoderm as well as the proper formation of mature cardiomyocytes.

### *Ccbe1* KD decreases the proliferation of differentiating ESCs

**Fig 5:**
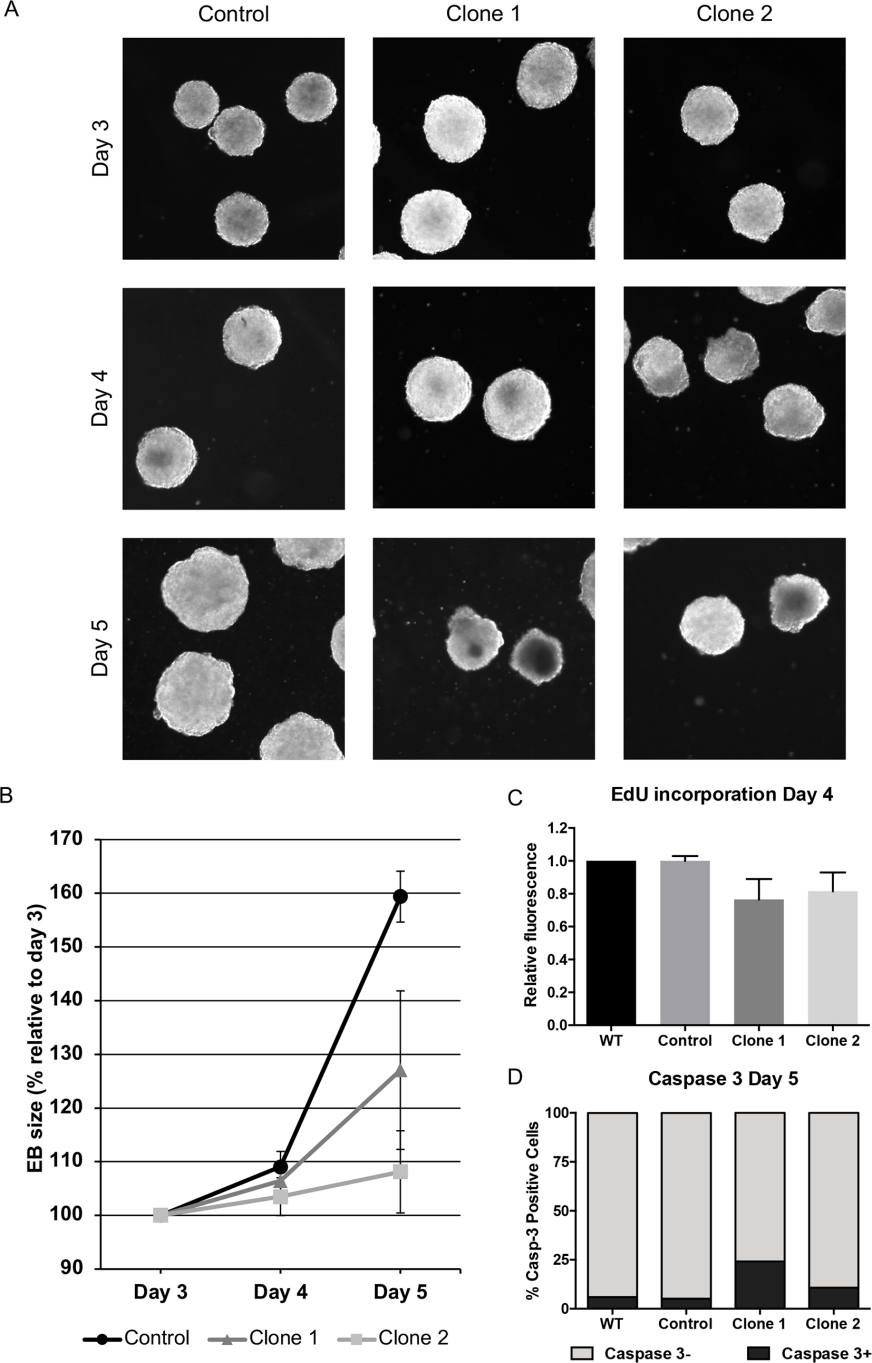
Ccbe1 loss-of-function leads to smaller embryoid bodies. (A) Phase-contrast micrographs of Ccbe1 KD ESC-derived embryoid bodies and control at days 3, 4 and 5 of differentiation. (B) Corresponding diameter increase of embryoid bodies at days 4 and 5 of differentiation (relative to day 3). (C) Cell proliferation was analyzed by labeling newly synthesized DNA using EdU incubation for 1.5 hours. Subsequent EdU labeling was done following the Click-iT EdU Alexa Fluor 488 Imaging Kit indications. Relative fluorescence intensity of cells was analyzed by flow cytometry. Minimum number of events: 20,000. (D) Quantification of immunolabelled CASP-3 positive cells analyzed by Flow cytometry at day 5 of differentiation.

Morphological analysis of the embryoid bodies revealed that both clones grew less in relative size between day 3 and 5 when compared to the control line (Fig 5A and 5B). We thus addressed the cellular mechanism causing the impaired growth of the *Ccbe1* KD ESC-derived embryoid bodies. Increased cell death and/or reduced proliferation are two probable causes that could explain the smaller relative size.

Next, we performed a proliferation assay consisting of labelling newly synthesized DNA with the thymidine analog EdU and posterior fluorescence labeling using the Click-iT EdU Alexa Fluor 488 Imaging Kit and analyzing the fluorescence intensity dependent on cell proliferation at day 4. This data strongly suggests that knockdown of CCBE1 results in a reduction in the proliferation of progenitors of differentiating *Ccbe1* KD ESC lines. This observation is consistent with the observed smaller size of the embryoid bodies derived from both *Ccbe1* KD clones (Fig 5C). Taken together, these data indicate that knockdown of *Ccbe1* leads to decreased proliferation of differentiating ESCs.

To understand whether the knockdown of *Ccbe1* also affects cell viability in differentiating ESCs, we evaluated caspase-3 mediated apoptosis and cell proliferation. Quantification of the percentage of apoptotic cells by immunolabelling against cleaved caspase-3 followed by FACS at day 5 of differentiation confirmed that there were a slightly bigger percentage of caspase-3 positive cells in clone 1 (21.1%) and clone 2 (10.8%) compared to WT and control cells (5.98 and 5.19% respectively) (Fig 5D). This suggests that both cell death and decreased proliferation could explain the smaller size of the embryoid bodies in the absence of *Ccbe1*.

These two results indicate that Ccbe1 affects both proliferation and cell death leading to a decreased embryoid body size.

### Treatment with CCBE1-blocking antibody during ESC differentiation mimics the defects induced by the knockdown

**Fig 6:**
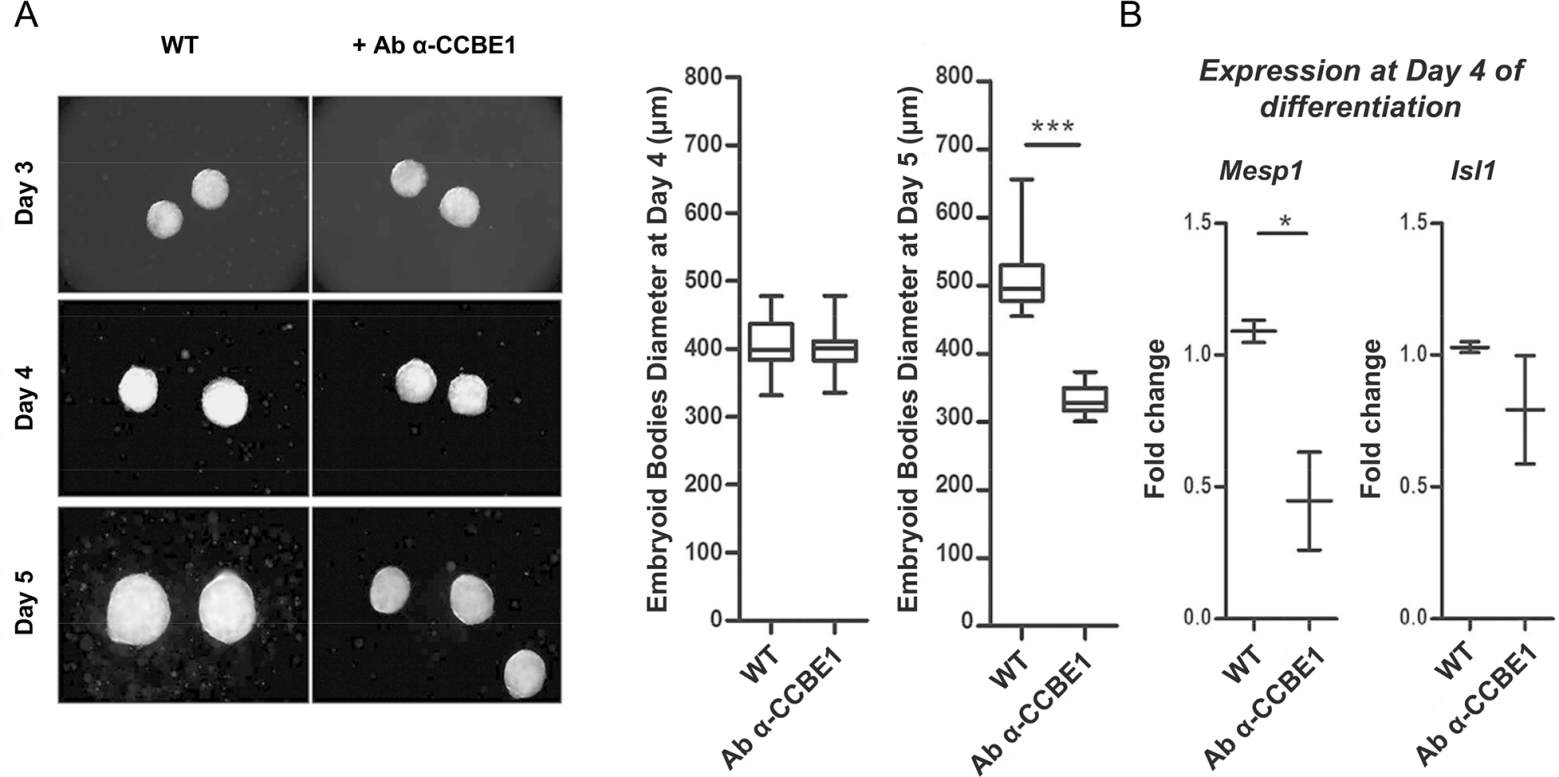
Ccbe1 loss-of-function by neutralizing antibody also leads to smaller embryoid bodies. (A) Phase-contrast micrographs of WT ESCs derived embryoid bodies at days 3, 4 and 5 of differentiation with and without supplementation of 100ng/mL of CCBE1 antibody. Graph on the right side shows the corresponding diameter measurements of the embryoid bodies at day 5 of differentiation; unpaired t-test relative to non-supplemented group. (B) qPCR analysis of cardiac precursor markers Mesp1 and Isl1at day 4 of differentiation. Data represent the mean ± SEM of three biological replicates in technical qPCR triplicates; paired t-test relative to Control group; statistical significance *p<0.05, **p<0.01, ***p<0.001.

To confirm that the absence of *Ccbe1* leads to the observed phenotype in our knockdown clones, we treated the WT RG ESCs during differentiation with CCBE1-blocking antibody to inhibit CCBE1 activity. ESCs were cultured up to day 5 of differentiation in the presence of 100 ng/mL CCBE1 antibody in the differentiation medium or with the equivalent amount of buffer as a control. As shown in Fig 6A, treatment with CCBE1 antibody led to a significant decrease on the size of the embryoid bodies derived from WT ESCs by day 5 of differentiation. Furthermore, of *Mesp-1 and Isl-1* expression at day 4 in differentiating ESCs treated with CCBE1 antibody was also downregulated (Fig 6B) similar to *Ccbe1* KD ESCs (Fig 4). This is consistent with the phenotype observed in the differentiating ESCs being specifically caused by *Ccbe1* loss-of-function.

## Discussion

Here we show that, like mouse and chick embryonic cardiac development [2, 3], *Ccbe1* is upregulated in SHF cardiac progenitors derived from ESC differentiating *in vitro*. Thus, high *Ccbe1* expression is correlated with the onset of cardiac specification both *in vivo* and *in vitro*. In addition, disruption of normal CCBE1 activity by shRNA knockdown or by blocking antibody results in a clear decrease in the expression of the early cardiac mesoderm markers *Mesp1* and *Isl1* at day 4. These results show that CCBE1 is required for the normal development of early cardiac precursors.

The developmental fate of differentiating ESCs depends on embryoid body size, growth factor signaling, ECM proteins that constitute the developmental niche and how ESCs interact with this niche [19–24]. Our results show that, in the absence of *Ccbe1*, the growth of differentiating embryoid bodies was arrested especially from day 4 onwards. This impaired embryoid body growth is caused by reduced cell proliferation and probably also by relative cell death. Therefore, it is possible that the reduced proliferation rate of *Ccbe1* KD ESCs differentiating in vitro could influence final cardiomyocyte formation. Nevertheless, the size of the embryoid bodies treated with CCBE1 blocking antibody was preserved at day 4 and was only slightly reduced at day 5. In contrast, the expression of the cardiac precursor markers was markedly diminished at day 4. This indicates that the cardiac mesoderm commitment defect is directly *Ccbe1* dependent and not primarily related to the size of the embryoid bodies. The effect of reduced embryoid body size seems to be more critical from day 5 onwards, coinciding with the time that cell proliferation is more severely affected and embryoid bodies are considerably smaller. From this time point onwards most alterations in gene expression may be related to the reduced size of the embryoid bodies and consequent alterations in the microenvironment of the cells. Therefore, our data suggests that, besides its role during early cardiac commitment, *Ccbe1* may have an independent role in the proliferation of differentiating ESCs.

In mice, *Ccbe1* loss-of-function results in embryonic lethality at E14.5 due to lymphangiogenesis defects [4]. Indeed, CCBE1 has been implicated in the modulation of VEGF-C signaling during mammalian lymphangiogenesis [4, 5] and zebrafish lymphangiogenesis [8, 25, 26] by promoting the *in situ* maturation of pro-VEGF-C into a mature lymphangiogenic-promoting form, thereby activating the downstream signaling pathway via VEGFR3 [7, 8].

However, only later in the in vitro differentiation process of ES cells (day 18), supplementation of mature VEGF-C has been shown to influence a lymphangiogenesis-like process [27, 28]. Therefore, this suggests that those target genes induced during lymphangiogenesis may not be activated by VEGF-C signaling or, alternatively, that the ESCs at early differentiation stages (days 4–8), as in our experimental set-up, do not respond to VEGF-C signaling. Altogether, this could indicate that during early ESC differentiation towards the cardiac lineage, CCBE1 may work through an unidentified mechanism independent of the VEGF-C/VEGFR3 pathway.

*In vivo*, *Ccbe1* is expressed at later stages of mammalian cardiac development [2], and both chick embryos and HS patients present cardiac defects when *CCBE1* is disrupted [3, 9–11]. Therefore, it would be interesting to study if *Ccbe1* has a role at later stages of ESCs cardiac differentiation. One possibility would be to study the disruption of the gene at specific time points later during cardiac commitment in differentiating ESCs. This would allow us to evaluate whether the modulation of *Ccbe1* can contribute *in vitro* to the full maturation of cardiac progenitors and/or beating cardiomyocytes.

Taken together, our data indicates that during ESC-derived cardiac differentiation *Ccbe1* is required to promote the formation of cardiac mesoderm precursor (*Mesp1*-positive) and progenitor (*Isl1*-positve) cells, and later on the differentiation of cardiomyocytes.

## Supporting information

S1 Table Primers for qPCR gene expression analysis. (DOCX)

S1 Fig Ccbe1 knockdown does not seem to affect normal Brachyury expression pattern between days 0 and 6. qPCR analysis at days 0, 2, 4 and 6 of the general mesoderm marker brachyury (*BraT*). Analysis was performed in two individual Ccbe1 KD ESC clones (Clone 1 and Clone 2) and relative expression is compared to day 0 of each cell type. Mean ± SEM of three biological replicates in technical qPCR triplicates.
(TIF)

## References

[1] Bento M, Correia E, Tavares AT, Becker JD, Belo JA. Identification of differentially expressed genes in the heart precursor cells of the chick embryo. Gene Expr Patterns. 2011;11(7):437–47. 10.1016/j.gep.2011.07.002 . 21767665

[2] Facucho-Oliveira J, Bento M, Belo JA. Ccbe1 expression marks the cardiac and lymphatic progenitor lineages during early stages of mouse development. Int J Dev Biol. 2011;55(10–12):1007–14. 10.1387/ijdb.113394jf . 22252499

[3] Furtado J, Bento M, Correia E, Inacio JM, Belo JA. Expression and function of Ccbe1 in the chick early cardiogenic regions are required for correct heart development. PLoS One. 2014;9(12):e115481 10.1371/journal.pone.0115481 . 25545279 PMC4278723

[4] Bos FL, Caunt M, Peterson-Maduro J, Planas-Paz L, Kowalski J, Karpanen T, et al CCBE1 is essential for mammalian lymphatic vascular development and enhances the lymphangiogenic effect of vascular endothelial growth factor-C in vivo. Circ Res. 2011;109(5):486–91. 10.1161/CIRCRESAHA.111.250738 . 21778431

[5] Hagerling R, Pollmann C, Andreas M, Schmidt C, Nurmi H, Adams RH, et al A novel multistep mechanism for initial lymphangiogenesis in mouse embryos based on ultramicroscopy. EMBO J. 2013;32(5):629–44. 10.1038/emboj.2012.340 . 23299940 PMC3590982

[6] Roukens MG, Peterson-Maduro J, Padberg Y, Jeltsch M, Leppanen VM, Bos FL, et al Functional Dissection of the CCBE1 Protein: A Crucial Requirement for the Collagen Repeat Domain. Circ Res. 2015;116(10):1660–9. 10.1161/CIRCRESAHA.116.304949 . 25814692

[7] Jeltsch M, Jha SK, Tvorogov D, Anisimov A, Leppanen VM, Holopainen T, et al CCBE1 enhances lymphangiogenesis via A disintegrin and metalloprotease with thrombospondin motifs-3-mediated vascular endothelial growth factor-C activation. Circulation. 2014;129(19):1962–71. 10.1161/CIRCULATIONAHA.113.002779 . 24552833

[8] Le Guen L, Karpanen T, Schulte D, Harris NC, Koltowska K, Roukens G, et al Ccbe1 regulates Vegfc-mediated induction of Vegfr3 signaling during embryonic lymphangiogenesis. Development. 2014;141(6):1239–49. 10.1242/dev.100495 . 24523457

[9] Alders M, Hogan BM, Gjini E, Salehi F, Al-Gazali L, Hennekam EA, et al Mutations in CCBE1 cause generalized lymph vessel dysplasia in humans. Nat Genet. 2009;41(12):1272–4. 10.1038/ng.484 . 19935664

[10] Connell FC, Kalidas K, Ostergaard P, Brice G, Murday V, Mortimer PS, et al CCBE1 mutations can cause a mild, atypical form of generalized lymphatic dysplasia but are not a common cause of non-immune hydrops fetalis. Clin Genet. 2012;81(2):191–7. 10.1111/j.1399-0004.2011.01731.x . 22239599

[11] Connell F, Kalidas K, Ostergaard P, Brice G, Homfray T, Roberts L, et al Linkage and sequence analysis indicate that CCBE1 is mutated in recessively inherited generalised lymphatic dysplasia. Hum Genet. 2010;127(2):231–41. 10.1007/s00439-009-0766-y . 19911200

[12] Burger NB, Bekker MN, Kok E, De Groot CJ, Martin JF, Shou W, et al Increased nuchal translucency origins from abnormal lymphatic development and is independent of the presence of a cardiac defect. Prenat Diagn. 2015;35(13):1278–86. 10.1002/pd.4687 . 26338284

[13] Jakus Z, Gleghorn JP, Enis DR, Sen A, Chia S, Liu X, et al Lymphatic function is required prenatally for lung inflation at birth. J Exp Med. 2014;211(5):815–26. 10.1084/jem.20132308 . 24733830 PMC4010903

[14] Domian IJ, Chiravuri M, van der Meer P, Feinberg AW, Shi X, Shao Y, et al Generation of functional ventricular heart muscle from mouse ventricular progenitor cells. Science. 2009;326(5951):426–9. 10.1126/science.1177350 . 19833966 PMC2895998

[15] Keller GM. In vitro differentiation of embryonic stem cells. Curr Opin Cell Biol. 1995;7(6):862–9. . 8608017 10.1016/0955-0674(95)80071-9

[16] Laugwitz KL, Moretti A, Caron L, Nakano A, Chien KR. Islet1 cardiovascular progenitors: a single source for heart lineages? Development. 2008;135(2):193–205. 10.1242/dev.001883 . 18156162

[17] Mommersteeg MT, Dominguez JN, Wiese C, Norden J, de Gier-de Vries C, Burch JB, et al The sinus venosus progenitors separate and diversify from the first and second heart fields early in development. Cardiovasc Res. 2010;87(1):92–101. 10.1093/cvr/cvq033 . 20110338

[18] Zhou B, von Gise A, Ma Q, Rivera-Feliciano J, Pu WT. Nkx2-5- and Isl1-expressing cardiac progenitors contribute to proepicardium. Biochem Biophys Res Commun. 2008;375(3):450–3. 10.1016/j.bbrc.2008.08.044 . 18722343 PMC2610421

[19] Zeng D, Ou DB, Wei T, Ding L, Liu XT, Hu XL, et al Collagen/beta(1) integrin interaction is required for embryoid body formation during cardiogenesis from murine induced pluripotent stem cells. BMC Cell Biol. 2013;14:5 10.1186/1471-2121-14-5 . 23350814 PMC3562267

[20] Bratt-Leal AM, Carpenedo RL, McDevitt TC. Engineering the embryoid body microenvironment to direct embryonic stem cell differentiation. Biotechnol Prog. 2009;25(1):43–51. 10.1002/btpr.139 . 19198003 PMC2693014

[21] Czyz J, Wobus A. Embryonic stem cell differentiation: the role of extracellular factors. Differentiation. 2001;68(4–5):167–74. . 11776469 10.1046/j.1432-0436.2001.680404.x

[22] Goh SK, Olsen P, Banerjee I. Extracellular matrix aggregates from differentiating embryoid bodies as a scaffold to support ESC proliferation and differentiation. PLoS One. 2013;8(4):e61856 10.1371/journal.pone.0061856 . 23637919 PMC3630218

[23] Higuchi S, Lin Q, Wang J, Lim TK, Joshi SB, Anand GS, et al Heart extracellular matrix supports cardiomyocyte differentiation of mouse embryonic stem cells. J Biosci Bioeng. 2013;115(3):320–5. 10.1016/j.jbiosc.2012.10.004 . 23168383 PMC5330950

[24] Taylor-Weiner H, Schwarzbauer JE, Engler AJ. Defined extracellular matrix components are necessary for definitive endoderm induction. Stem Cells. 2013;31(10):2084–94. 10.1002/stem.1453 . 23766144 PMC3812380

[25] Astin JW, Haggerty MJ, Okuda KS, Le Guen L, Misa JP, Tromp A, et al Vegfd can compensate for loss of Vegfc in zebrafish facial lymphatic sprouting. Development. 2014;141(13):2680–90. 10.1242/dev.106591 . 24903752

[26] Hogan BM, Bos FL, Bussmann J, Witte M, Chi NC, Duckers HJ, et al Ccbe1 is required for embryonic lymphangiogenesis and venous sprouting. Nat Genet. 2009;41(4):396–8. 10.1038/ng.321 . 19287381

[27] Liersch R, Nay F, Lu L, Detmar M. Induction of lymphatic endothelial cell differentiation in embryoid bodies. Blood. 2006;107(3):1214–6. 10.1182/blood-2005-08-3400 . 16195336 PMC1895915

[28] Mishima K, Watabe T, Saito A, Yoshimatsu Y, Imaizumi N, Masui S, et al Prox1 induces lymphatic endothelial differentiation via integrin alpha9 and other signaling cascades. Mol Biol Cell. 2007;18(4):1421–9. 10.1091/mbc.E06-09-0780 . 17287396 PMC1838981

