# Supplementary material for: Loss of Ccbe1 affects cardiac-specification and cardiomyocyte differentiation in mouse embryonic stem cells
Source: PLoS One. 2018 Oct 3;13(10):e0205108. doi: 10.1371/journal.pone.0205108 (PMC6169972; doi:10.1371/journal.pone.0205108)
Supplement: S1 Table — (DOCX) [file pone.0205108.s001.docx]

| **Gene** | **Forward (5’–3’)** | **Reverse (5’–3’)** |
| --- | --- | --- |
| **α*Mhc*** | GATGGCACAGAAGATGCTGA | CTGCCCCTTGGTGACATACT |
| ***Ccbe1*** | ATGGGACCTATGGGACCTTC | AGTGAGTCCGGTGTCCAAAC |
| ***cTnt*** | GGAAATCCAAGATCACTGCCTCC | GGGCACTGAGGGACAGACCA |
| ***Gapdh*** | GGGAAGCCCATCACCATCTTC | AGAGGGGCCATCCACAGTCT |
| ***Isl1*** | CCTGTGTGTTGGTTGCGGCA | GGGCACGCATCACGAAGTCG |
| ***Mesp1*** | TGTACGCAGAAACAGCATCC | TTGTCCCCTCCACTCTTCAG |
| ***Nkx2.5*** | CCACTCTCTGCTACCCACCT | CCAGGTTCAGGATGTCTTTGA |
| ***Pgk1*** | ATGGATGAGGTGGTGAAAGC | CAGTGCTCACATGGCTGACT |
