# Supplementary figures and images for: Loss of Ccbe1 affects cardiac-specification and cardiomyocyte differentiation in mouse embryonic stem cells
Source: PLoS One. 2018 Oct 3;13(10):e0205108. doi: 10.1371/journal.pone.0205108 (PMC6169972; doi:10.1371/journal.pone.0205108)

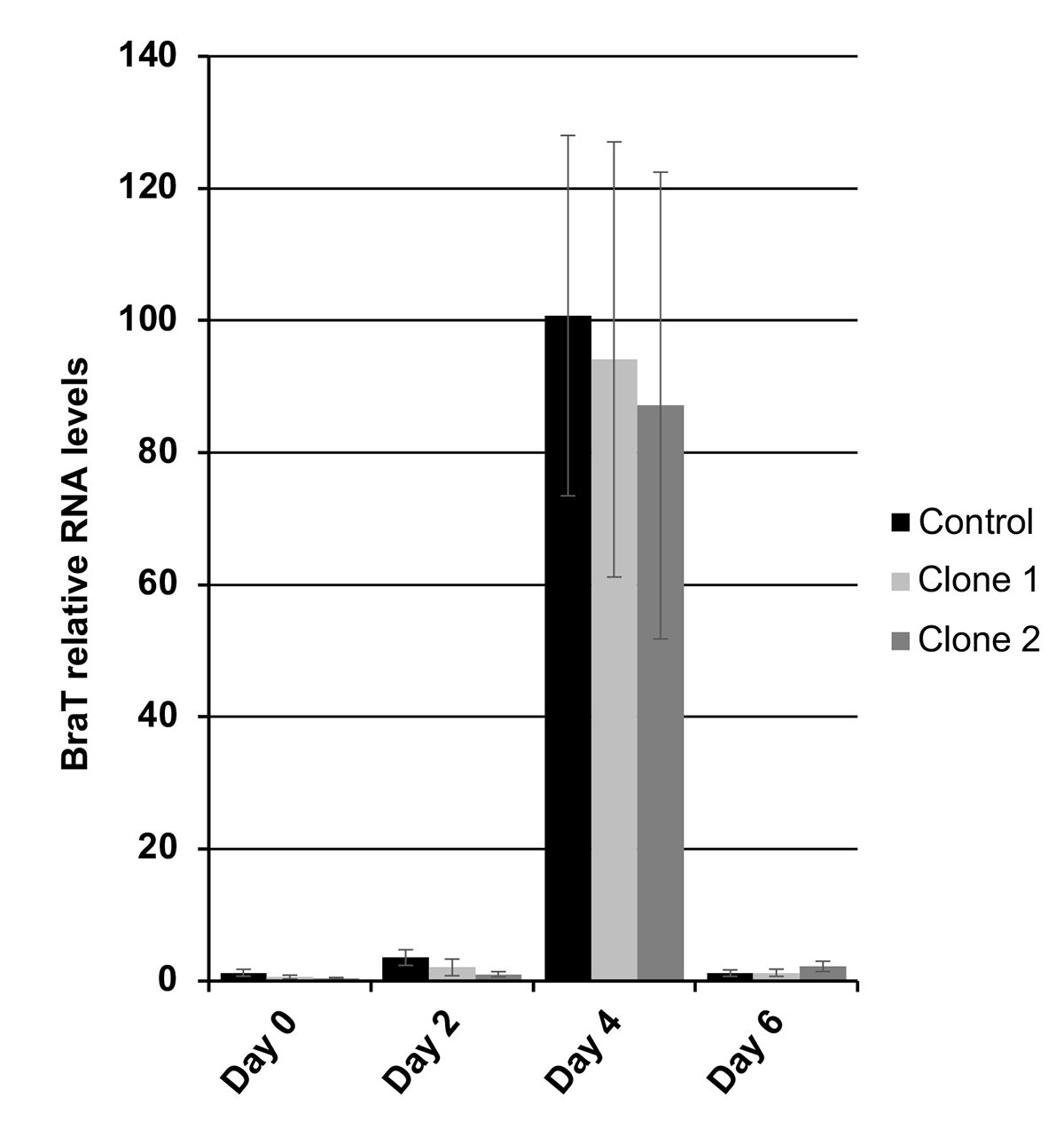

Supplement: S1 Fig — qPCR analysis at days 0, 2, 4 and 6 of the general mesoderm marker brachyury (BraT). Analysis was performed in two individual Ccbe1 KD ESC clones (Clone 1 and Clone 2) and relative expression is compared to day 0 of each cell type. Mean ± SEM of three biological replicates in technical qPCR triplicates. (TIF) [file pone.0205108.s002.tif]
